# We Cannot Put This Genie Back in the Bottle: Qualitative Interview Study Among Family Medicine Providers About Their Experiences With Virtual Visits During the COVID-19 Pandemic

**DOI:** 10.2196/43877

**Published:** 2023-08-31

**Authors:** Saskia T Spiess, Elena Gardner, Cindy Turner, Annie Galt, Katherine Fortenberry, Tiffany Ho, Jordan Knox, Dominik Ose

**Affiliations:** 1 Department of Family and Preventative Medicine School of Medicine University of Utah Health Salt Lake City, UT United States

**Keywords:** family medicine, primary care, virtual visits, telemedicine, COVID-19

## Abstract

**Background:**

When a genie is freed from its bottle, things cannot be restored to the way they were before. At the beginning of the global COVID-19 pandemic, health care systems adjusted how they delivered care overnight. Primary care practices switched from seeing patients in person to virtual care applications, including video and phone visits, e-visits, e-consults, and messaging with clinicians. Prior to the pandemic, these applications were not as widely used, but discussions around their advantages and disadvantages in some settings were being explored. Emergency regulatory changes spurred by the pandemic freed this virtual care “genie” from its bottle. Wide-scale adoption of virtual care in family medicine has much potential, as primary care services are often a patient’s first point of contact with the health care system.

**Objective:**

This study aims to analyze family medicine providers’ experiences using virtual visits during the pandemic, perceived outcomes of the shift to virtual visits, and discusses its implications for the future of family medicine.

**Methods:**

This qualitative study took place at 3 academic primary care clinics between June and December 2020. Data were collected through one-on-one Zoom (version 5.2.1) interviews with family medicine clinical faculty who experienced the rapid transition of in-person visits to mostly “virtual” visits. The interviews were recorded, deidentified, and transcribed. We adopted a constructivist approach to qualitative content analysis to evaluate the results.

**Results:**

In total, 25 participants were eligible, and 20 individuals participated in this study (80% participation rate). The mean age was 43.4 years, and 85% (17/20) of the participants were female. We identified 3 main themes: the care process, patient engagement, and team-based care.

**Conclusions:**

This study highlights the transition from in-person to virtual visits during the pandemic from the perspective of family medicine providers. Generally, family medicine providers’ perceptions of the shift to virtual visits were positive, especially regarding team-based care. Challenges involved virtual inhibition, particularly for providers. Providers described ways they integrated virtual care with aspects of in-person care, creating a hybrid environment. The genie is out of the bottle—things will not be the same—but family medicine now has the opportunity to evolve.

## Introduction

In early 2020, at the beginning of the global COVID-19 pandemic, a genie escaped its bottle as health care systems adjusted how they delivered care overnight. Primary care practices rapidly transitioned from in-person to remote care [1,2]. In the first months of the pandemic, virtual encounters increased by 766% [3]. Now, virtual care applications, defined as video and phone visits, e-visits, e-consults, and messaging with clinicians [4], have been tested in extreme circumstances. While overall usage rates have since decreased and stabilized, virtual care is now standard practice [3]. However, once a genie is out of the bottle, things cannot be restored to the way they were before.

Virtual care is not a new method for delivering health care services. Before the pandemic, virtual care visits were effective conduits for treating patients with anxiety and depression, increasing patient access to specialists, and providing personalized chronic disease management [5-8]. However, widespread adoption of virtual care beyond phone visits was slow, typically attributed to strict regulatory infrastructure [9-11]. The literature on virtual care predicted dramatic implications for the health care system after wide-scale adoption, including reduced health care costs, substantial expansion of access to high-quality health care, and several challenges [4,9,10].

In particular, primary care and family medicine practices have much to gain from virtual care integration. They are often the first entry point into the health care system, provide referrals to specialists, and consistently support patients with chronic illnesses, behavioral health concerns, and substance use disorders [12-14]. Primary care will continue to have a critical role in managing the broader and long-term consequences of the pandemic [15].

Today, there is a need for enhancing evidence and establishing best practices for virtual care processes, applications, and care coordination to better prepare for the standard application of virtual care and a possible next disaster or emergency [1,16]. While care has largely shifted back from being virtual to being in-person in the 2 years since the onset of the pandemic, there are also signs of a hybrid model of care delivery emerging [4]. The hybrid model of care reflects the effective integration of both in-person and virtual care. As said by Dorn, “to unlock the full potential of virtual care, health care systems must make it a central element of care design” [4]. The hybrid structure of care delivery has already been shown to be beneficial in various settings [17-19].

Patient perceptions of telehealth services, before and after the onset of the pandemic, are widely documented [20-22]. Regarding providers, an early pandemic scoping review found positive perceptions of telehealth among a variety of health care providers [23]. Another scoping review of mostly internal medicine providers also had largely celebratory findings [24]. However, less is known about how family medicine providers, in particular, perceived the shift to virtual visits during the pandemic and the lasting impacts of that shift on family medicine practice. Therefore, this study aimed to describe family medicine providers’ experiences with virtual visits during the pandemic, perceived outcomes of the shift to telemedicine, and discuss its implications for the future of medicine.

## Methods

### Study Design

This qualitative study took place at the University of Utah Health System between June and December 2020. We used a constructivist approach for this study due to the exploratory nature of the topic. Interviews, or one-on-one conversations about the transition from “in-person” to mostly “virtual” visits, were the preferred method in this study. This individuality allowed participants more privacy due to the subject matter and minimized interparticipant influence. The interviews were conducted virtually to help minimize barriers to participation, support a higher response rate, and adhere to COVID-19 safety guidelines still in place at the time of the study.

### Study Sample and Recruitment

Eligible participants were clinical faculty from the 3 University of Utah academic Family Medicine health centers (Madsen, Sugarhouse, and Centerville). Family medicine clinical faculty who used virtual visits because of COVID-19 were eligible for this study. In this study, clinical faculty included Family Medicine clinicians and a clinical psychologist, referred to as “providers,” or “family medicine providers” in the results and discussion. Specialists and other health care providers who did not use virtual visits because of COVID-19 or do not practice family medicine were excluded from the study. Participants were recruited via email and verbal announcements within faculty-wide meetings. In line with recommendations for nonprobabilistic sampling, we estimated that between 15 and 20 interviews would be needed to reach saturation [25]. In total, 25 participants were eligible, and 20 individuals participated in this study (80% participation rate).

### Data Collection

The research team developed a semistructured interview guide using qualitative research principles to facilitate the interviews. The questions addressed provider’s experiences with virtual visits, particularly the relationship between provider and patient. The interviews were conducted virtually via Zoom and lasted about 30 minutes. Interviews were audio and visual recorded and then transcribed. Participants completed a survey after the interview to collect demographic data.

### Data Analysis

Qualitative content analysis was used to evaluate the results of the interviews. First, the transcribed interviews were analyzed independently by several researchers (STS, DO, and CT) and coded by category. Categories were added and edited when new information came to light. Once the researchers established categories, they were organized into themes and subthemes and supported by evidence from the transcripts. The research team expanded to 5 individuals who then discussed these themes and subthemes until they reached a consensus regarding the results.

### Focus and Definitions

For this data analysis, we focused on the care process, patient relationships, and professional collaboration. We defined the “care process” as the workflow surrounding patient care, from the initial contact between the health care team and patient, until the end of the visit. “Professional collaboration” is more than one provider working with others toward a common patient health goal. Finally, the “patient relationship” is defined as all interactions between the health care faculty and the patient, which may impact adherence to treatment guidelines. The implementation of virtual visits with short notice at the start of the pandemic presented multiple challenges and opportunities concerning the care process and patient relationship; hence we defined our subcategories accordingly as “challenges” and “opportunities” for the “care process” and “patient relationship.” Subcategories for “team-based care” were “virtual integration of care” and “versatility of care,” reflecting the lack of challenges in this category.

### Ethical Considerations

The University of Utah’s institutional review board undertook a human subject research ethics review (IRB #00133384) and exempted this study. Informed consent was obtained from all participants, and participation in the interview was considered consent to include responses in the study. Participants’ responses were deidentified to protect privacy. There was no compensation for participation in this study.

## Results

### Presentation of Results

For reader friendliness, the tables summarize each thematic group by category and the main findings for each category. In addition, selected quotations are mentioned in the body text. An overview of all relevant quotations and how they informed results can be found in Multimedia Appendices 1-3.

### Participant Characteristics

Table 1 describes the cohort. A total of 20 providers were interviewed, including a psychologist (n=1) and physicians (n=19) with various clinical specialties. The mean age was 43.4 years, and 85% (17/20) of the participants were female. On average, participants have been health care professionals for 14.2 years, but individual time spent practicing varies. Most participants spend at least half of their time in the clinic.

**Table 1:** Cohort description (N=20).

Characteristics			Values
Age (years), mean (range)			43.4 (29-67)
Sex (female), n (%)			17 (85)
**Job type, n (%)**			
	Psychologists		1 (5)
	**Family medicine physicians**		19 (95)
		Subspecialty^a^	5 (25)
		No subspecialty	15 (75)
Years in practice, mean (range)			14.2 (1-39)
**Full-time equivalent^b^, n (%)**			
	Full-time		5 (25)
	Half-time		15 (75)

^a^Additional fellowship training or subspecializations reported include: geriatrics (n=1), obstetrics (n=1), addiction medicine (n=1), bariatrics (n=1), and sports medicine (n=1).

^b^Full-time equivalent defined as >50% time in clinic, half-time equivalent defined as ≤50% time in clinic.

### Care Process

The shift to virtual visits revealed methods for improving the care process, but not without challenges. Challenges were caused by the inability to implement standard care procedures virtually; for example, performing a virtual physical examination is impossible. Table 2 summarizes the challenges and opportunities relating to a shift to virtual visits. Physicians adapted to fewer physical examination methods and identified several ways virtual visits could improve health outcomes for certain patients.

**Table 2:** Care process.

Category	Result
Challenges	Cannot perform physical examinations virtually Cannot follow a traditional care model Not possible to collect laboratory specimens virtually More difficult to prescribe a new medication or diagnose a new condition More difficult to treat substance abuse Potentially more difficult to manage chronic conditions virtually
Opportunities	Increase opportunities for behavioral health interventions Improve patient adherence Increase flexibility in communication Improve virtual chronic care with basic equipment

#### Care Process Challenges

Under the traditional care model, as taught during medical school education, a patient with diabetes would participate in a physical examination during their clinic visit (eg, annual foot examination) and have to collect laboratory samples for analysis (eg, blood work to measure hemoglobin A_1c_ levels). In the virtual care environment, however, providers cannot do these things, causing health care providers to become worried they might mismanage care after not having access to data typically used in clinical decision-making. Another concern was the challenge of diagnosing a new condition virtually and feeling discomfort when prescribing a new medication in some uncertain scenarios.

There were also challenges in managing existing conditions. A lack of evidence-based guidelines on the virtual management of chronic illnesses fostered concerns by the faculty that they may not always provide the highest level of care.

We’re used to practicing medicine that’s evidence based, and there’s not a lot of evidence basis for video visits, [...] I’m afraid [...] of missing something [...] or [...] that I’m not always doing the right thing for my patient by doing a video visit.

I19: 382-384

Furthermore, substance use disorder treatment makes up a large proportion of primary care visits, especially in our university-based clinics. Providers identified 2 main challenges in offering virtual substance use disorder treatment, represented in the quote below: (1) inability to instantaneously monitor laboratory testing results and (2) challenges in building the patient-doctor relationship virtually.

Suboxone treatment [...] I really do like to have people come in [...] to do the labs that are necessary. [...] That patient doctor relationship is so important [...] and [...] I can do that better in person.

I6: 123-125

#### Care Process Opportunities

Although providers identified challenges in the virtual care process, they also identified many unique opportunities. Providers felt that virtual care allowed increased opportunities for behavioral health interventions. Behavioral health treatment can often pose unique challenges, and patients cancel in-person appointments for various reasons.

Allowing providers to address these concerns virtually effectively reduces barriers to care and could improve patient adherence to scheduled visits. In the virtual model, even when a patient forgets they had a scheduled visit, connecting and ensuring a patient improves with prescribed treatment is still possible if the medical assistant (MA) can reach the patient.

Providers also liked having the opportunity to address more complex questions with a virtual visit instead of communicating via “messaging platforms.”

I really like the flexibility of if someone asks me a question, a long, detailed question via our messaging platform, through My Chart, and I can just say, listen, this needs a virtual visit, let’s get this done, I can see you at two o’clock today.

I20: 270-272

Many providers receive messages from patients regarding their care that are very complex. Before the virtual care process, these questions would be addressed by a telephone call, after hours or during lunch breaks, or the provider would schedule an appointment 1-2 weeks later. With the virtual platform, a provider can often schedule the patient that same day due to last-minute no-shows or cancellations, have a more meaningful conversation, and ensure all questions are addressed. An unintended benefit of virtual visits is that the provider does not need to stay in the clinic after hours but can address concerns during their scheduled clinic time.

While another provider commented chronic disease management could be a challenge, another highlighted the potential to improve the virtual care process using basic equipment. The provider noted patients’ successful self-monitoring at home and reported blood pressure and weight for virtual visits. They suggest an improvement to the virtual care approach could include patients collecting data points at home, such as using a scale or a blood pressure cuff.

### Patient Relationship

Providers identified both challenges and opportunities to strengthen their relationships with patients in the virtual environment. Table 3 outlines those challenges and opportunities. Patients and providers needed to adjust to new forms of communication during virtual visits, and providers were able to involve more aspects of the patient’s lived experience in the visits.

**Table 3:** Patient relationship.

Category	Results
Challenges	Providers may miss nonverbal cues (input) Providers unable to express empathy nonverbally (output) Patients experience virtual inhibition too
Opportunities	Insight into patient’s home environment Strengthen family or patient involvement Intensify or deepen patient or provider relationship

#### Patient Relationship Challenges

The major challenge providers identified regarding the patient relationship is “virtual inhibition.” We coined this term to describe the inability of providers to express nonverbal empathy to patients and the challenge of identifying nonverbal cues from patients. Some providers felt that a comprehensive encounter with a patient included interaction with the entire body, not just the forehead.

I think that there are things that you can share with a person that are unspoken, like [...] Emotional cues, [...] that can only really happen when you can [...] interact with somebody’s whole body. [...] I’m trained to interact with somebody’s whole body, not just their forehead.

I7:175-178

The above example describes some providers’ frustration interacting with their patients on a screen, which typically only depicts the face. This perspective was challenging to some providers, who rely on body posture and other bodily cues when gathering their history of present illness to aid in their clinical decision-making.

Other providers felt virtual inhibition when expressing empathy; for example, when a patient was upset in person, the provider might have offered a hug to comfort them. This gesture is not feasible in the virtual environment, leading many to feel like they were unable to build adequate rapport with patients. Furthermore, as building patient rapport throughout a clinic visit shapes much of the ongoing patient-doctor relationship, this could challenge treatment adherence and future interactions.

#### Patient Relationship Opportunities

Nevertheless, providers identified many opportunities for improving the patient relationship in virtual care settings (Table 3). Virtual care takes the patient out of the examination room and places them in a more familiar environment. Observing how patients move and act in their own space uniquely intensifies the provider-patient relationship. Providers can gain insights into aspects of the patient’s life they may have otherwise been unaware of or neglected. Some felt they gained insight into what is essential to their patient by seeing their home environments, such as background paintings and family photos. Using artwork became a way to start a conversation and connect with patients on a meaningful level, ultimately helping them feel safe enough to disclose medical concerns they may not have disclosed otherwise.

Also, the involvement of family members and caretakers became much easier in the virtual environment, particularly in scenarios where adult children that wanted to be involved in their elderly parent’s care lived in a different location. In the past, adult children were hindered from participating in their parent’s appointments due to work, physical distance, or life circumstances. They can now connect virtually through an email or text link to join the appointment. This opportunity is described well in the quote below:

One patient of mine that I see on zoom, whose daughter gets on zoom, [...], I was trying to explain [...] that she was going to go have to see a neurosurgeon [...] her daughter included the brother [...] on the zoom call.

I5: 268-272

The above example demonstrates how family members living in different parts of the country can participate in one another’s care remotely, thus overcoming prior barriers to family involvement. This involvement allows the family to have a more meaningful conversation on recommended treatment approaches, which, in this instance, could be invasive and require neurosurgery consultation. Other family members at the visit can help listen to treatment options and discuss them together when the patient is ready.

### Team-Based Care

The patient-provider relationship is crucial, but providing high-quality patient care is not a solo endeavor; it relies on an entire team. Table 4 summarizes the aspects of virtual integration of care and versatility of care presented as opportunities for virtual visits. In this context, virtual integration refers to how different care team members can work together “external” to the clinic; for example, the clinician and clinical pharmacy team may not be in the same space but can collaborate with each other and the patient on a virtual platform. Versatility of care refers to the team “internal” to the clinic, comprised of the front desk staff, MA, and clinician.

**Table 4:** Team-based care.

Category	Results
Virtual integration of care (external to clinic)	Combined care team or PCP^a^ visit Integrated specialist consult Integrated ancillary services (home health and physical therapy)
Versatility of care (internal to clinic)	Improved collaboration between MAs^b^ and providers Increased provider flexibility in workflow Increased MA flexibility

^a^PCP: primary care provider.

^b^MA: medical assistant.

#### Virtual Integration of Care (External to Clinic)

Participants identified the virtual integration of care teams and a specialist as advantageous. For example, one provider captured the opportunity of the external integration of care in a team-based visit with a behavioral health team and a primary care provider (PCP).

I think what would be [...] great is thinking about how we use this in [...] care conferences. And I did it recently where [...] we had behavioral health care management and pharmacy on the phone. And [...] I was the physician lead.

I6: 312-314

As shown in the example above, there is care integration between primary care, behavioral health, and pharmacy teams. The PCP looped in the entire team during one visit rather than having to schedule multiple follow-up visits with all the team members.

Additionally, in the example below, providers were able to coordinate with ancillary services more efficiently. Patients had home health aides and nurses physically evaluate a wound, for example, with the provider virtually present, and then develop a combined treatment plan. Not only was care integrated, but a barrier to communication of care was eliminated as the provider could directly communicate a treatment plan with the home health nurse.

It’s a lot easier to get home health and so we actually would do his visits when home health came so that she could undress the wound and I could look at it.

I5: 137-138

#### Versatility of Care (Internal to Clinic)

Professional collaboration within the clinic also evolved through virtual care. Participants repeatedly pointed out that providers and MAs benefited from flexibility within the schedule. MAs could control the workflow more efficiently and help keep providers’ schedules running on time. For example, an MA could check in a virtual patient earlier if an in-person patient ran late. Then, the provider could see the virtual patient first instead of waiting for the late patient and avoid running behind schedule for the rest of the session.

I think MAs kind of like it because [...] they can work ahead and keep you on time. [...] we have a different level of control over the ability to keep the schedule going with virtual visits. [...] if they spend a lot of time trying to get my 2:30 patient on the phone, and they can’t. [...] for some reason [...]. I can see my three o’clock patient, [...] and then I can go back and recall my earlier patient.

I5: 245-250

However, not only MAs appreciated having more control over the schedule. Providers also liked that they could see patients earlier and rearrange their schedules more effectively.

The example below shows improved provider satisfaction with their workflow and more control over their schedules.

My life is not as dictated by the schedule. If there’s issues with someone connecting, I can call them later and maybe see someone else sooner that I couldn’t do that in person.

I5: 258-259

Another interesting result was improved versatility in the workflow. While MAs would still ask standard “check-in” questions, providers shifted away from the once common use of templates (known as x-files, within the study setting’s system, each “chief complaint” was associated with a standardized set of “history of present illness” questions that our MAs completed). This shift allowed MAs to focus on other important patient care tasks, such as helping address telephone encounters and patient messages.

So, they [MAs] are ensuring that the patients are set up on the technology appropriately. And then they’re still asking them questions. [...] we’re doing much more abbreviated versions [...] not the full sort of x file questionnaire before.

I5:195-196

## Discussion

### Overview

In summary, our study described family medicine provider experiences and perceived outcomes of the shift to virtual visits during the COVID-19 pandemic. Providers identified 3 main areas of challenges and opportunities in virtual care delivery: the care process, the patient-provider relationship, and team-based care.

### Care Process

First, family medicine providers identified several opportunities for improving the care process through virtual visits, including the potential to improve chronic care management, improved patient adherence from decreased physical barriers, and increased flexibility and communication throughout the care process. At the same time, family medicine providers identified challenges associated with the abrupt and uncertain transition to virtual visits, namely replicating established physical procedures in the virtual environment and a lack of established best practices.

Literature about how family medicine providers perceive the impact of virtual visits on the care process is less common than literature about health care providers in general or other specialties of medicine [15,26-28]. Based on the experiences in other specialties, virtual visits’ impact on the care process has been met with mixed feelings from providers [26]. For example, in one systematic review of primary care and mental health services, virtual consultations were as effective as in-person, and patient satisfaction with virtual visits was high. However, providers noted it may not be the best option for all patients [15]. At the same time, one study in an ambulatory hospital setting shows providers like virtual visits less than patients and that providers fear widening disparities among vulnerable populations such as older populations, populations with low health literacy, or low-income populations [27]. Finally, echoing the concerns of family medicine providers in this study, shared concern about performing effective and acceptable assessments or diagnoses during virtual visits [28].

### Patient-Provider Relationship

Next, regarding the patient-provider relationship, family medicine providers described what we coined “virtual inhibition,” or a lack of human touch and connection, as a major challenge. Providers worried they would miss nonverbal cues from the patient and struggled to express nonverbal empathy. Despite these challenges, providers felt that virtual visits provide an opportunity to strengthen family involvement and shared decision-making in patient care by removing barriers like physical distance. Providers also described how seeing a patient’s home environment strengthened their relationship with the patient.

Echoing challenges with “virtual inhibition” during virtual visits from the participants in this study, a letter from later in the pandemic reflected on how challenging it was to offer empathy to patients in a time where hugs or hand-holding was not permitted, and nonverbal empathy was communicated almost exclusively through the eyes when everyone masked up [29]. However, similarly to the care process, there is less information on family medicine providers’ relationship with their patients during this shift than in other medical specialties [30,31]. For example, in one study, patients and PCPs disagreed regarding virtual visits’ impacts on their relationship. Patients felt that the virtual environment influenced the provider’s attentiveness. In contrast, and similar to our findings, providers appreciated the insight into patients’ home lives and felt it strengthened the relationship [30]. Participants in this study shared similar feelings about gaining a better picture of a patient’s home environment through virtual visits. Finally, although not from the provider perspective, one report prior to the pandemic claimed that patients feel virtual visits strengthen the relationship with their PCP because they are able to keep their provider familiar with their condition through virtual visits [31].

### Team-Based Care

Finally, family medicine providers described how the shift to virtual visits impacted team-based care with other health care providers and within the clinic. First, participants described how virtual integration of care promoted interdisciplinary collaboration and identified combined care team visits, integrated specialist consultations, and collaboration with ancillary services like home health, physical therapy, or pharmacy as opportunities to improve care. Second, participants described how virtual visits improve team-based care through increased versatility in the clinic. Participants felt positive about virtual visits as they increased collaboration between providers and MAs and increased flexibility among staff and faculty alike.

While there is a lack of literature on virtual visits for team-based care in family medicine practice, there is research from other health care professionals on this subject [32-34]. One systematic review conducted prior to the pandemic discussed care for patients with cancer and found multidisciplinary teams to be valuable. They argue that the inception of multidisciplinary teams resulted in better management of and improved quality of life for patients with cancer because of active discussion and a better understanding of how treatments can be combined across specialties to optimize patient outcomes [32]. The authors also make a case for integrating care teams into clinical practice (and recommend teleconferencing to accomplish this in some settings) [32], echoing the potential opportunities for virtual integration of care identified by participants in this study. Similar calls for integration of care have been made regarding diabetes self-management and management of multiple chronic conditions [33,34].

### Implications for the Future of Family Medicine

Before the pandemic, conversations about a new care paradigm were emerging. “Care 4.0” happens when technology enables the right care at the right time, and the health care system adapts to patient’s changing needs by providing flexible engagement with services. This type of flexible engagement has also been described as creating “a community of care” where technology does not replace in-person connections but facilitates connections by enabling informal carriers and other services to participate in care together with formal health care services [35].

The COVID-19 pandemic entirely changed how medicine and virtual care are delivered, practiced, and taught. Looking to the future, family medicine, concerned largely with improving health and care throughout the life course and recognizing biopsychosocial determinants of health, is especially well poised to adopt new models that increase accessibility and acceptability of health care services to a variety of patients. Our study showed that family medicine providers generally perceive virtual visits as an opportunity to improve the delivery of care, quality of care, and patient satisfaction—not without challenges, of course.

Other areas in medicine have already shown that the hybrid model works for providers and patients. Potential changes to the care process identified by participants in this study have been attempted in areas outside of family medicine and have been successful for patients and providers, and they all involve a hybrid option [36-38]. Regarding the patient-provider relationship, with patients managing chronic conditions in particular, continuity of care that takes advantage of the virtual environment can help manage chronic conditions. Not only are patients with chronic conditions likely to use virtual visits, but they like maintaining a preexisting relationship [39]. During the pandemic, interdisciplinary collaboration was encouraged. Successful examples of that collaboration included team-based care for diabetes and cardiovascular health, the crucial role of pharmacists on teams, and the inclusion of medical language interpreters in a hybrid capacity, and all resulted in positives for the patient [28,34,40,41].

### Limitations

One limitation of this study is that participants were recruited from one university-based health system in one state, which could limit the generalizability of the results. This limitation is mitigated by participants working across several clinic locations and serving a broad range of patients within the health system. Additionally, participants were interviewed in 2020 during the initial onset of the pandemic, and technology has continued to evolve since then. Finally, participants were predominantly female. Future research should include perspectives from male, transgender, and nonbinary family medicine providers.

Researchers and providers should note that virtual visits’ success will depend on equitable access to the infrastructure necessary to support telephone or video visits and comprehensive patient or provider education and training to increase digital literacy. Additionally, establishing best practices and policies for virtual care will be required to take full advantage of opportunities to improve patient care and help incorporate virtual care as a permanent part of our health care delivery system.

### Conclusions

This study highlights the transition from in-person to virtual visits during the pandemic from the perspective of family medicine providers. In general, family medicine providers’ perceptions of the shift to virtual visits were positive, especially regarding team-based care. Providers described ways they integrated virtual care with aspects of in-person care, creating a hybrid environment. The genie is out of the bottle—things will not be the same—but family medicine now has the opportunity to evolve based on the lessons of the pandemic.

## Appendix

Multimedia Appendix 1 Care process.

Multimedia Appendix 2 Patient-provider relationship.

Multimedia Appendix 3 Team-based care.

## Abbreviations

MA: medical assistant
PCP: primary care provider

## Data Availability

The data sets generated and analyzed during this study are available from the corresponding author upon reasonable request. Complete interviews are not publicly available to protect the providers’ privacy within the health system.
