# Supplementary material for: We Cannot Put This Genie Back in the Bottle: Qualitative Interview Study Among Family Medicine Providers About Their Experiences With Virtual Visits During the COVID-19 Pandemic
Source: J Med Internet Res. 2023 Aug 31;25:e43877. doi: 10.2196/43877 (PMC10502598; doi:10.2196/43877)
Supplement: Multimedia Appendix 1 [file jmir_v25i1e43877_app1.docx]

| Category | Result | Supporting Quotes |
| --- | --- | --- |
| Challenges | Cannot perform physical exam virtually | *You can’t do a physical exam [...] I listen and I touch. (I15: 392-393)* |
|  | Not possible to collect laboratory specimens virtually | *It's a lot easier to get labs done [...] after the visit when they're there in person. But when [...] you have to remember [...] to go in and get your blood drawn, it doesn’t always happen. (I18: 301-303)* |
|  | Harder to prescribe a new medication or diagnose a new condition | *I'm much more reluctant to start a new medication or make a new diagnosis via a virtual visit than I am in person. (I20: 190-191)* |
|  | Harder to treat substance abuse | *Suboxone treatment [...] I really do like to have people come in [...] to do the labs that are necessary. [...] That patient doctor relationship is so important [...] and [...] I can do that better in person (I6: 123-125)* |
|  | Cannot follow a traditional care model | *We're used to practicing medicine that's evidence based, and there's not a lot of evidence basis for video visits, [...] I'm afraid, afraid of missing something [...] or [...] that I'm not always doing the right thing for my patient by doing a video visit. (I19: 382-384)* |
|  | Potentially harder to manage chronic conditions virtually | *I think a lot of chronic diseases probably aren't taken care of as well [...] with diabetes [...] they can't really do a home A1C. [...] I can't do a foot exam at home. (I16: 157-164)* |
| Opportunities | Increase opportunities for behavioral health interventions | *I think virtual visits work really well for a lot of mood related concerns. Because you don't really usually [...] have to do much of a physical exam in the first place. (I18: 244-248)* |
|  | Improve patient adherence | *It's easier, and maybe you have less no shows a little bit on that too, because it's easier to track people down because they're just wherever they are, instead of having to actually get into clinic. (I18: 244-248)* |
|  | Increase flexibility in communication | *I really like the flexibility of if someone asks me a question, a long detailed question via our, our messaging platform, through My Chart, and I can just say, listen, this needs a virtual visit, let's get this done, I can see you at two o'clock today. (I20: 270-272)* |
|  | Improve virtual chronic care with basic equipment | *I've talked to some of my patients about getting a scale and a blood pressure cuff. I'm even talked to a few about getting an oximeter, but I have them weigh themselves and check their blood pressure before the visit [...] then I just order the labs and have them go get them in a university lab that's convenient at the end of the visit. (I11: 156-159)* |
