# Supplementary material for: We Cannot Put This Genie Back in the Bottle: Qualitative Interview Study Among Family Medicine Providers About Their Experiences With Virtual Visits During the COVID-19 Pandemic
Source: J Med Internet Res. 2023 Aug 31;25:e43877. doi: 10.2196/43877 (PMC10502598; doi:10.2196/43877)
Supplement: Multimedia Appendix 2 [file jmir_v25i1e43877_app2.docx]

| Category | Results | Supporting quotes |
| --- | --- | --- |
| Challenges–“Virtual inhibition” | Patients experience virtual inhibition too | *I think people are less inclined to open up about [...] how they're really feeling [...] I had a patient [...] tell me that she didn't want to do virtual therapy anymore because she didn't feel like she could really tell her therapist what was going on with her over a zoom call. (I7: 153-156)* |
|  | Providers may miss nonverbal cues (input) | *I think that there are things that you can share with a person that are unspoken, like [...] Emotional cues, [...] that can only really happen when you can [...] interact with somebody's whole body. [...] I'm trained to interact with somebody's whole body, not just their forehead. (I7: 175-178)* |
|  | Providers unable to express empathy nonverbally (output) | *In person would be better [...] in an empathic moment, [...] I might hug a patient who's really in distress and say, [...] may I give you a hug, I can see you're really upset or I might touch them. (I6: 159-161)* |
| Opportunities | Insight into patient’s home environment | *When people talk to me from home, [...] I asked them [...] like, oh,[...], tell me about the art over your shoulder.... [...] I use the environment that I'm seeing as a way of connecting on a personal level. (I6: 176-178)* |
|  | Strengthen family or patient involvement | *One patient of mine that I see on zoom, whose daughter gets on zoom, [...], I was trying to explain [...] that she was going to go have to see a neurosurgeon [...] her daughter included the brother [...] on the zoom call. (I5: 268-272)* |
|  | Intensify or deepen patient or provider relationship | *I get to see people on this even more personal capacity than before, and [...] get a different sense of their lives, then, I [...] would have ever had the insight in prior to this. [...] I sort of love that aspect of it (I5: 149-151)* |
