# Supplementary material for: We Cannot Put This Genie Back in the Bottle: Qualitative Interview Study Among Family Medicine Providers About Their Experiences With Virtual Visits During the COVID-19 Pandemic
Source: J Med Internet Res. 2023 Aug 31;25:e43877. doi: 10.2196/43877 (PMC10502598; doi:10.2196/43877)
Supplement: Multimedia Appendix 3 [file jmir_v25i1e43877_app3.docx]

| Category | Results | Supporting quotes |
| --- | --- | --- |
| Virtual Integration of Care (External to Clinic) | Combined care team or primary care provider (PCP) visit | *I think what would be [...] great is thinking about how we use this in [...] care conferences. And I did it recently where [...] we had behavioral health care management and pharmacy on the phone. And [...] I was the physician lead. (I6: 312-314)* |
|  | Integrated specialist consult | *Could see some really great collaboration between [...] primary care and a specialist [...] if you had to do some sort of [...] care conference of getting multiple specialists in a room together, we could do this now. (I5: 286-289)* |
|  | Integrated ancillary services (home health, physical therapy) | *It’s a lot easier to get home health and so we actually would do his visits when home health came so that she could undress the wound and I could look at it. (I5: 137-138)* |
| Versatility of Care (Internal to Clinic) | Improved collaboration between Medical Assistants (MAs) and providers | *I think MAs kind of like it because [...] they can work ahead and keep you on time. [...] we have a different level of control over the ability to keep the schedule going with virtual visits. [...] if they spend a lot of time trying to get my 2:30 patient on the phone, And they can't. [..] for some reason [...]. I can see my three o'clock patient, [...] and then I can go back and recall my earlier patient. (I5: 245-250)* |
|  | Increased provider flexibility in workflow | *My life is not as dictated by the schedule. If there's issues with someone connecting, I can call them later and maybe see someone else sooner that I couldn't do that in person. (I5: 258-259)* |
|  | Increased MA flexibility | *So they [MAs] are ensuring that the patients are set up on the technology appropriately. And then they're still asking them questions. [...] we’re doing much more abbreviated versions [...] not the full sort of x file questionnaire before. (I5: 195-196)* |
